# Motivation and planning as mediators of the relation between social support and physical activity among U.S. adolescents: a nationally representative study

**DOI:** 10.1186/1479-5868-11-42

**Published:** 2014-03-21

**Authors:** Kaigang Li, Ronald J Iannotti, Denise L Haynie, Jessamyn G Perlus, Bruce G Simons-Morton

**Affiliations:** 1 Health Behavior Branch, Eunice Kennedy Shriver National Institute of Child Health & Human Development, 6100 Executive Blvd, Bethesda, MD 20892-7510, USA; 2 University of Massachusetts Boston, 100 Morrissey Blvd, Boston, MA 02125-3393, USA

**Keywords:** Physical activity, Motivation, Planning, Social support, Adolescents, SDT

## Abstract

**Background:**

More than half of U.S. high-school students do not meet the moderate and vigorous physical activity (MVPA) 5 hours per week recommendation. The purpose of this study was to determine how individual dimensions (motivation and planning) mediate the relationship of social context with physical activity by integrating available measures of personal characteristic including internal/external motivations (derived from Self-Determination Theory -SDT]) for MVPA, MVPA planning, peer MVPA, and parental support to better understand adolescent MVPA.

**Methods:**

Survey responses of a nationally representative cohort of 11^th^ graders (N = 2439) in the NEXT Generation Health Study were analyzed with structural equation modeling.

**Results:**

Adolescent MVPA was directly, significantly associated with MVPA planning (*β* = 0.17), peer MVPA (*β* = 0.21), and internal motivation (*β* = 0.50). Internal motivation was associated with peer MVPA (*β* = 0.31), parental support for MVPA (*β* = 0.16), and external motivation (*β* = 0.40). A significant relation between parental support and external motivation (*β* = 0.31) was also found.

**Conclusions:**

Adolescents with higher internal motivation and more active friends were more likely to engage in MVPA. The results are consistent with SDT and suggest that planning is an important construct for adolescent MVPA.

## Background

The benefits of regular physical activity (PA) for adolescents include enhanced physical, psychological/mental, and social well-being [1,2]. Yet, there is still a large portion of adolescents who have not engaged in sufficient PA in the US. For example, in 2011 more than half of US high-school students engaged in PA less than 60 minutes/day on 5 or more days a week [3], the threshold in adolescence for decreasing the odds of obesity in subsequent adulthood [4]. Identifying and understanding determinants of PA are prerequisites for successfully promoting PA engagement among adolescents.

Motivation is essential for purposeful action, including PA [5]. Many theories of motivation [6,7] and motivation-related constructs [8,9] have been examined to explain the goal-directed behavior of PA. In particular, Self-Determination Theory (SDT) provides a well-validated framework for understanding the dynamics of motivation for the initiation and maintenance of PA [10]. However, SDT is best considered within a social context because SDT posits that different kinds of specifiable social-contextual factors may either facilitate or hinder one’s innate tendency towards a behavior [11] such as PA. For instance, relatedness facilitates motivated behavior [11]. Therefore, social influence from friends and parents may play critical roles in enhancing or diminishing adolescents’ motivation for and engagement in PA. Although motivation is critical for people to be internally driven to act, Bandura and Simon [12] argue that either intention or desire alone cannot significantly affect behavior if one does not have the capacity for exercising influence over his/her own motivation. In this sense, action planning by the self should be necessary as a bridge between motivation and behavior. The review of literature will further articulate those constructs and likely interplay.

### Self-determination and motivation

SDT conceptualizes motivation along an intrinsic-extrinsic continuum [10]. Intrinsic motivation describes autonomously organized and regulated behavior. People seek novelty and challenge, and are intrinsically motivated to act in ways that are inherently satisfying. Thus the perceived source or cause of intrinsic motivation is internal to the person. In contrast, extrinsic motivation is perceived as being in response to some externally-imposed demand but has the potential to be internalized. Accordingly, behavior could occur due solely to external rewards and punishments, or the internalization and valuing of external regulation and behavioral goals [11]. Amotivation describes when one is neither internally nor externally motivated to engage in the behavior.

### PA planning

Action planning (the act of consciously scheduling and/or arranging to engage in a behavior) may serve as a necessary bridge between motivation and behavior [11,13]. Recent studies have found that planning is a good predictor of PA [13-15]. Although a large number of studies have examined the association between different types of motivation [10], regulation [16], and PA [17-19], PA planning has not been clearly examined as a mediator of the relation between motivation and PA.

### Social influence

Social context and social support are important influences on motivation, particularly with respect to how people interpret external factors [11]. Research indicates that social support from friends [20] and family members [21] is associated with higher levels of PA. Vallerand [22] proposed that social factors stimulate one’s external and internal motivation for, and persistence in, participating in PA. Peer and parental influences have been studied in association with motivation for and participation in PA. For example, in one study, the mere presence of peers and friends was found to increase youths’ motivation to engage in PA [23]. Conceivably, the association between adolescent and peer PA could be due to injunctive peer norms (perceptions about how peers want the adolescent to behave) [24], support [25], modeling [25], and peer selection and socialization processes [25,26]. Furthermore, internal motivation for PA has been found to partially mediate the relation between peer social support and PA [27].

Research findings on parents’ influence on youth’s engagement in PA are mixed. Some studies found that positive parenting practices, either in general or specific to PA, predicted engagement in PA. For example, Ornelas and colleagues [25,28] found that family cohesion, parent–child communication, and parental engagement positively predicted moderate to vigorous PA for both genders (grades 7 to 13) one year later, and King and colleagues [26] found that parental encouragement to exercise predicted more frequent engagement in PA in the past week for high school students from freshmen to seniors. In contrast, a cross-sectional study did not find an association between parent support and youth (ages 10–17) PA, although there was association between parent self-PA and youth PA [29]. The inconsistent results require more studies on parent influence on adolescent PA.

### Current study

**Figure 1:**
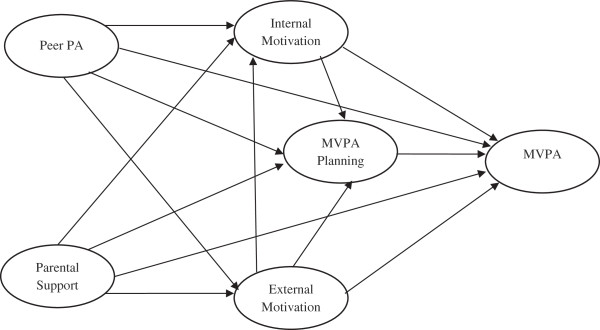
Hypothesized associations between internal/external motivations for moderate and vigorous physical activity (MVPA), MVPA planning, peer PA, parental support, and adolescent MVPA.

This study addresses some of the inconsistencies regarding the relations between SDT constructs and youth PA. The purpose of this study is to examine a hypothesized model (Figure 1), with particular interest in the role of planning. The following research hypotheses were examined: (1) favorable intrinsic and extrinsic motivations are associated with more PA engagement; (2) PA planning mediates the relationship between motivation and PA engagement; and (3) positive social environment, specifically peer PA and parental support, relates to adolescents’ increased PA engagement, motivation for PA engagement, and PA planning.

## Methods

### Sampling

This cross-sectional analysis examines data from the 11^th^ grade assessment (Wave 2) of the NEXT Generation Health Study, a nationally representative probability cohort study. School districts, the primary sampling unit (PSU), were stratified by the nine Census divisions. Within each Census division, the sample of PSUs was first selected with probability proportional to the total enrollment. A total of 137 schools with 10^th^ grade students were randomly recruited and 81 (response rate = 64%) agreed to participate. Classes were randomly selected within each participating school and 3796 students were recruited to participate. Of those students, 2619 students (response rate = 69%) provided assent and their parents provided consent. A total of 2439 11^th^ grade students (55% [weighted] female) completed a survey during the 2010–2011 academic year (Wave 2). For students who turned 18 in the year between assessments, consent was obtained. African-American participants were oversampled to provide better population estimates and to provide an adequate sample to examine racial/ethnic differences. The study protocol was reviewed and approved by the Institutional Review Board of the *Eunice Kennedy Shriver* National Institute of Child Health and Human Development.

### Measures

#### Physical Activity (PA)

PA was measured with two closed-ended questions. One asked how many of the past 7 days (options 0 to 7 days) they were physically active for a total of at least 60 minutes per day, derived from a validated question in the Youth Risk Behavior Surveillance survey [30]. Examples of PA, such as running, brisk walking, rollerblading, biking, dancing, skateboarding, swimming, soccer, basketball, football, & surfing were provided immediately before this question. The second asked how many hours a week they usually engage in vigorous PA, defined as "any activity that increases your heart rate and makes you get out of breath some of the time", (response options 1 = none, 2 = about a half hour, 3 = one hour, 4 = 2–3 hours, 5 = 4–6 hours, and 6 = 7 hours or more), derived from a validated question in the Health Behavior in School-Aged Children (HBSC) survey [31]. Each of these questions collected information on moderate and/or vigorous PA.

To examine demographic differences in preliminary analyses, the scores of the first question were dichotomized to determine percentage of students meeting the recommended 60 minutes/day on at least 5 days a week [3,4]. The dichotomous variable was used for preliminary analysis. For all other analyses, a latent variable comprised of the two questions was created, with all the response categories of the first question used.

#### Internal and external motivation

Internal and external motivations for MVPA were measured with scales developed for this study. Based on SDT [5,32], the internal motivation scale consisted of three items which map onto intrinsic, integrated and identified regulations, respectively: (1) I enjoy it; (2) It fits with how I see myself; and (3) It is personally important to me. The external motivation scale maps onto non-regulation, external and introjected regulations: (1) I am required to do it; (2) My parents, other family members, or friends tell me to do it; and (3) I feel guilty if I do otherwise. All items were measured with options 1 = not at all true to 7 = very true. For the current sample, the internal consistency coefficient of internal motivation was 0.84 and that of external motivation was 0.60.

#### MVPA planning

Planning for PA was measured with three items derived from Dombrowski [15]. The participants were asked how often in the last seven days they planned for vigorous PA including (1) when to exercise; (2) how often to exercise; and (3) where to exercise (response options 1 = not at all to 5 = very often). For the current sample, the internal consistency coefficient of this scale was 0.93.

#### Peer physical activity

Peer physical activity (Peer PA) was measured by three items derived from the National Longitudinal Study of Adolescent Health [33] by asking participants how often they thought their closest male friend, closest female friend, and five closest friends did vigorous PA at least three times a week with options from 1 = never to 5 = almost always. The internal consistency coefficient of this scale was 0.74 for the current sample.

#### Parental support for MVPA

A single item derived from the National Survey on Drug Use and Health [34] was used to measure student perceived parental support for daily MVPA and or exercise by asking participants how important it was to your parents/guardians that you get daily MVPA and/or exercise, with response options from 1 = not at all to 7 = extremely. Higher scores reflect higher levels of parental support.

#### Demographic and other potential control variables

Participants reported age, gender, racial/ethnic background, and family socioeconomic status. Parents provided education levels of both parents at the time they signed informed consent. Parent education was categorized as less than high school diploma, high school diploma/GED, some college/technical school/advanced degree, and bachelors/graduate degree. Family socioeconomic status was estimated using the Family Affluence Scale [35] including number of cars owned (0 = No, 1 = Yes, 1 and 2 or more = Yes) and computers owned (0 = None, 1 = One, 2 = Two, and 3 = More than two), whether the student had his/her own bedroom (0 = No and 1 = Yes), and the number of family vacations in the last 12 months (0 = Not at all, 1 = Once, 2 = Twice, and 3 = More than twice). Based on the infrequency of responses in the highest categories for owning computers and family vacations, both variables were recorded so that the highest category equaled two or more. Students were categorized as low (0, 1, 2, 3, or 4), moderate (5 or 6) or high affluence (7) based on the total score (from 0 to 7) [36].

#### Statistical analysis

Exploratory factor analysis (EFA) was used to identify the scales measuring proposed latent variables and confirmatory factor analysis (CFA) was used to test whether the data fit a hypothesized measurement model. Structural equation modeling (SEM) was used to test both direct and indirect relations of latent constructs with the outcome variable (i.e., physical activity) simultaneously. Maximum likelihood parameter estimates with standard errors (MLR) was used as the estimator, which are robust to deviations from normality and non-independence of observations.

Model fit was assessed using (a) the Chi square statistic, (b) Standardized Root Mean Square Residual (SRMR), (c) Root Mean Square Error of Approximation (RMSEA), (d) the Comparative Fit Index (CFI) and (e) the Tucker–Lewis index (TLI) [36]. The following thresholds were used to determine model fit: a non-significant chi-square; a SRMR value below 0.10, a RMSEA less than 0.06, and CFI and TLI values approaching 1.0 [37].

Mediation was assessed using indirect, direct, and total effect [38] based on improved SEM approach [38,39]. Given an independent variable (X), a dependent variable (Y) and a potential mediator (M) in a SEM model, M is considered a mediator if there are direct significant effects on paths X → M and M → Y and indirect significant effect on the specific path X → M → Y, conditional on the presence of other mediators in the model. Total effect (X → M → Y) and direct effect (X → Y) are compared to assess the possible attenuated relationship between X and Y when accounting for the M. Wald test was used to compare parameter estimates (path coefficients) [40].

Statistical analyses were performed using SAS 9.2 and Mplus 7. Features of the complex survey design (i.e., stratification, clustering and sampling weights) were taken into account in the analyses. Standard errors were computed based on the multistage stratified design of the survey. We examined the intraclass correlation coefficient (ICC, the index indicating the proportion of variance in the outcome that is between groups) of PA by schools, school districts, and census divisions. The ICCs were fairly low (0.05 to 0.08), indicating that the variance in PA is mainly explained by students rather than higher-level variables.

## Results

Of the 2439 participants (*M* = 17.31 years and *SE* = 0.07), 55.0% (weighted, the same hereinafter) were females, 19.6% were Hispanic/Latino (vs. 17.6% African Americans, 58.6% Whites, and 4.3% other minorities), 21.9% were from low-affluence families (vs. 50.3% from moderate and 27.8% from high affluence families), and 8.2% of students had one parent with less than high school diploma as the highest education level (vs. 24.2% with high school diploma/GED, 40.5% with some college, teaching school, and advanced degree, and 27.2% with bachelors or higher degree).

### Preliminary results

As shown in Table 1, 48.6% (weighted, the same hereinafter) of study participants met the MVPA 5 hour/per week recommendation. Meeting this recommendation was more prevalent among males (61.8%) than females (37.6%), and among students with parents who had bachelors or higher degrees (57.6%) than those with parents who had less than high school diploma (41.2%), high school diploma (47.6%), and some college or similar degree (46.6%). African American students (38.2%) were less likely to have met the recommendation compared to White (52.1%), Hispanic (44.0%) and other (62.8%) students. No significant association was found between family affluence and meeting PA 5 hours/per week recommendation.

**Table 1:** Percent of students who met the MVPA 5 hour/per week recommendation by descriptive characteristics

	Meeting recommendation*			
	**N**	**Weighted%**	**Rao-Scott χ**^**2**^	***p***
Total	2427	48.62	--	--
Gender				
Male	1069	61.77	93.65	<0.001
Female	1350	37.63		
Race/Ethnicity				
Whites	976	52.05	11.39	0.01
Hispanic	707	44.04		
Black	603	38.24		
Other	118	62.78		
Family affluence				
High	479	49.14	2.07	0.35
Moderate	1031	51.11		
Low	654	44.63		
Education level, higher of both parents				
Less than high school diploma	286	41.21	8.00	<0.05
High school diploma/GED	511	47.55		
Some college/technical school/AD degree	765	46.59		
Bachelors/graduate degree	497	57.55		

*Note. At least 5 hours of MVPA a week was recommended to decrease the odds of obesity during adolescent [4].

### Multivariate results

Five factors were identified from fourteen items (the single item for parental support was not included) using the EFA model: MVPA (2 items), internal motivation (3 items), external motivation (3 items), MVPA planning (3 items), and peer PA (3 items) with clear rotated loadings for five factors and good values of fit indices [RMSEA (90% CI) = 0.044 (0.039 – 0.050); CFI = 0.965; TLI = 0.923; SRMR = 0.027]. The factor structure using 6 constructs (including the single item perceived parental support) was confirmed in a single model using confirmatory factor analysis technique with good values of fit indices [RMSEA (90% CI) = 0.030 (0.027 – 0.034); CFI = 0.972; TLI = 0.961; SRMR = 0.034].

**Figure 2:**
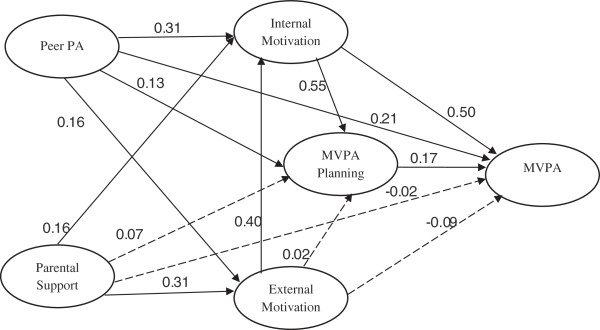
**Structural equation modeling to test an integrated model of adolescent physical activity.** RMSEA (90% CI) = 0.030 (0.027 – 0.034); CFI = 0.952; TLI = 0.927; χ^2^_df = 167_ = 446.542, *p* < 0.001; AIC = 94834.846; BIC = 95485.528; Adjusted BIC = 95110.646; SRMR = 0.035. Standardized parameter estimated; Dotted lines represent paths which are not significant; Demographic variables pointed to all latent variables but not shown. MVPA = moderate and vigorous physical activity.

The hypothesized model (Figure 1) was then tested and found to have an acceptable fit to the data. The direct paths among the key theoretical constructs were shown in Figure 2, with significant relations indicated by solid lines. Standardized coefficients were provided to make it easier to compare the magnitudes of relations via different paths.

As shown in Figure 2, MVPA planning was directly associated with adolescent MVPA. External motivation was directly associated with internal motivation but not with MVPA planning or MVPA. Internal motivation, however was directly associated with MVPA planning and MVPA. Peer PA was directly associated with internal and external motivation, MVPA planning, and MVPA. Parental Support was directly associated with internal and external motivation.

Peer PA was significantly but dissimilarly (Wald test value = 11.80, *p* < .001) associated with internal (*β* = 0.31) and external (*β* = 0.16) motivations. Similarly parental support also showed significant associations with internal (*β* = 0.16) and external (*β* = 0.31) motivation, although the difference was not significant (Wald test value = 1.31, *p* > .05).

The indirect relation (0.10, *p* < .01) of internal motivation with adolescent MVPA via MVPA planning was much smaller (Wald test value = 37.29, *p* < .001) than the direct relation (0.50, *p* < .01) of internal motivation with adolescent MVPA, which suggests the importance of internal motivation to engaging in MVPA. Notably, 83% variability in MVPA was accounted for by direct relation and 17% by indirect relation controlling for other factors in the model. Parental support was not significantly related to adolescent MVPA directly in either of the two models, but it was indirectly related through MVPA Planning.

Mediation of the relation between internal motivation and MVPA by MVPA planning was examined. As shown in Table 2, total effect and direct effect between internal motivation and MVPA were 0.60 (*p* < .001) and 0.50 (*p* < .001), respectively; the indirect effect between internal motivation and MVPA via planning was 0.10 (*p* < .01). The direct effect remained significant when MVPA planning was included as a mediator, indicating that MVPA planning partially mediated the relationship between internal motivation and MVPA. We also tested mediation of the relationship between external motivation and MVPA planning by internal motivation. Complete mediation was found such that the total effect between external motivation and MVPA planning was 0.24 (*p* < .001), the indirect effect between external motivation and internal motivation was 0.22 (*p* < .001), and the direct effect (0.02) was not significant (*p* > .05, Table 2).

**Table 2:** Standardized total, direct and indirect effects between latent variables

**Path**	**Standardized estimate**	**Standard error**
PA planning to PA		
Total	0.17^**^	0.07
Total indirect	0	0
Direct	0.17^**^	0.07
Internal Motivation (IM) to PA		
Total	0.60^***^	0.06
Total indirect	0.10^**^	0.04
Direct	0.50^***^	0.05
IM – planning – PA	0.10^**^	0.04
Internal Motivation (IM) to PA planning		
Total	0.55^***^	0.05
Total indirect	0	0
Direct	0.55^***^	0.05
External Motivation (EM) to PA		
Total	0.15^**^	0.06
Total indirect	0.24^***^	0.05
Direct	-0.09	0.06
EM – planning – PA	0.003	0.01
EM – IM – PA	0.20^***^	0.04
EM – IM – planning – PA	0.04^*^	0.02
External Motivation (EM) to PA planning		
Total	0.24^***^	0.05
Total indirect	0.22^***^	0.04
Direct	0.02	0.05
EM – IM – planning	0.22^***^	0.04
Peer PA to PA		
Total	0.44^***^	0.04
Total indirect	0.23^***^	0.04
Direct	0.21^***^	0.04
Peer PA – IM – PA	0.15^***^	0.03
Peer PA – planning – PA	0.02^*^	0.01
Peer PA – EM – planning – PA	<0.001	0.001
Peer PA – EM – IM – PA	0.03^***^	0.01
Peer PA – EM – IM – planning - PA	0.01^*^	0.003
Parental support (PS) to PA		
Total	0.13^***^	0.04
Total indirect	0.15^***^	0.03
Direct	-0.02	0.04
PS – IM – PA	0.08^***^	0.02
PS – IM – planning – PA	0.01^**^	0.01
PS – planning – PA	0.01	0.01
PS – EM –PA	-0.03	0.02
PS – EM – planning – PA	0.001	0.003
PS – EM – IM – PA	0.06^***^	0.02
PS – EM – IM – planning – PA	0.01	0.01

*p < .05, **p < .01, ***p < .001.

PA = moderate and vigorous physical activity.

## Discussion

In this study, nearly half of the participants reported meeting the recommended guideline of 60 minutes a day 5 days a week, although there was considerable variability. We examined associations with adolescent MVPA and internal and external motivation, MVPA planning, peer PA, and parental support. MVPA prevalence was higher among males than females, White than Hispanic or Black youth, and among moderate and higher family affluence and parent education compared to lower affluence and education, consistent with other research. Importantly, the findings are consistent with other literature and theory that suggest that MVPA planning is associated with MVPA [19,27]. Our findings support part of the first hypothesis that more PA engagement is directly associated with positive intrinsic motivation, but only indirectly with extrinsic motivation. The findings partially support our second hypothesis that MVPA planning mediates the relationship between internal motivation and MVPA engagement. Motivation was associated with planning for MVPA, such that internal motivation completely mediated the relation between external motivation and planning. Partial support was also found for our third hypothesis that features of the social environment were associated with adolescent MVPA. Peer PA was directly associated with adolescent MVPA, adolescent MVPA motivation, and adolescent MVPA planning (and thereby also indirectly related to adolescent MVPA through MVPA planning). Parental support was indirectly associated with MVPA, through its direct association with internal and external motivation.

Ryan and colleagues [17] posit that external motives such as losing weight and feeling more attractive may be important in initiating MVPA, but motivation driven by internal factors (e.g., enjoyment and competence) are more important for long-term adherence to MVPA. Ntoumanis reported a SEM analysis that showed that internal motivation was related to MVPA intention (a key prerequisite to performing a behavior) among adolescents. In contrast, external regulation and amotivation were not related to MVPA intention and were predictors of boredom while engaging in MVPA [16]. Our findings are consistent with previous research showing that internal motivation has a strong, positive, direct association with youth MVPA, and external motivation has a non-significant negative association. Given that our MVPA questions measured non-habitual MVPA behavior, the results suggest adolescents’ MVPA may be mainly the product of internal motivation.

Social influences can affect motivation positively or negatively, such that internal motivation for MVPA can be promoted through support and positive feedback from other people; but can also be undermined by undesired external pressure and control [10]. Adolescents may be particularly susceptible to social influence, given their relatively insufficiently developed decision making capabilities [41] and heightened reward sensitivity in the presence of peers [42]. Keegan and colleagues [43] found that peers and parents, among other social agents, can influence youth motivation and participation in sport. Specifically, parents fostered children’s motivation by support and facilitation, whereas peers influenced motivation and collaborative behaviors [43]. Our findings indicate that perceived peer PA and parental support were associated with both internal and external motivation for PA. Interestingly, the association between peer PA and internal motivation is stronger than the association between peer PA and external motivation. Adolescents may be more likely to perceive peer PA as an echo of their enjoyment in MVPA (internal motivation) than external pressure (external motivation). Alternatively, participation in MVPA with peers may contribute to the enjoyment of MVPA, and also serve to enhance peer relations, providing or reinforcing internal motivation [44]. This suggests that friends may play an important role in fostering adolescents’ adherence to a long-term MVPA regimen [10]. The comparable associations of parental support with internal/external motivation indicate that parents’ support of adolescents’ MVPA engagement may be perceived by adolescents as either as external pressure or encouragement of their inherent interest in MVPA engagement. However, if parental support of adolescents’ MVPA is perceived as pressure, its influence may be minimal given that adolescents’ external motivation is not linked to MVPA directly. Consistent with a recent systematic review, in this study adolescent’s MVPA was significantly associated with peer PA but not with parental support [45]. Parental encouragement of MVPA appears to decline between early and middle adolescence [46], possibly in relation to the increase in the salience of peer influence [47]. Additionally, although the direct association between parents’ support and MVPA was not significant, the indirect association via internal motivation was significant. This suggests that parents’ influence on adolescent MVPA may be more likely to be exerted through adolescents’ internal motivation than by only showing their expectation for children’s MVPA. It may also suggest the possibility that an important function of parenting with respect to adolescent PA is facilitation of planning. Future studies are needed to look into the mechanisms by which parents influence children’s PA MVPA.

We examined the mediation effect of MVPA planning on internal motivation and MVPA engagement and found a direct path between MVPA planning and MVPA among adolescents. In the current study, we also found significantly indirect association between internal motivation and MVPA via MVPA planning, although it was weaker compared to the direct association. Therefore, we believe MVPA planning may be an important intermediary to MVPA. Planning reflects the details of how, when, and how much MVPA and therefore reflects skill and competence at planning, which are known to derive from as well as to enhance motivation [11]. A previous study found self-regulation skills to be the most proximal predictor of MVPA behavior and mediator of the link between planning and MVPA in adults [13]. A recent study found that adolescents with greater planning skills were more likely to successfully translate their intentions into MVPA plans [48]. Therefore, it may be useful to include MVPA planning skills training in MVPA promotion programs.

The study has limitations. First, the lack of longitudinal data precludes examination of causal relationships between motivation, planning, and MVPA behavior [49]. It may also limit our ability of to identify changes in the relations between planning and engaging in MVPA. Sniehotta and colleagues [13] found that the effect of coping planning increased over time in cardiac patients. In this regard, future longitudinal studies are needed to investigate the prospective association between MVPA planning, internal/external motivation for MVPA, and MVPA engagement. Second, the school-based recruitment might limit the generalization of the findings to adolescents not in school. Third, the internal consistency coefficient of our three items of external motivation is relatively low. Fourth, the single-item measure of parental support on adolescents’ MVPA may limit the dimensions of the construct. Finally, our measures of MVPA did not differentiate between sport and exercise, and may have included some everyday activities such as active transportation. Each of these sources of MVPA may have somewhat different motivational origins.

## Conclusions

Adolescents who plan for MVPA are more likely to engage in MVPA, have greater internal motivation, and more active friends are more likely to engage in MVPA. Internal motivation, parent support, and peer PA were significantly associated with adolescent PA planning. Our findings are consistent with SDT concepts and suggest that MVPA planning may be important for adolescent MVPA. Longitudinal data are needed to clarify these interactive relationships over time and better inform interventions on adolescents’ MVPA. Planning may be an important construct that should be considered along with SDT.

## Abbreviations

MVPA: Moderate to Vigorous Physical Activity; SDT: Self-Determination Theory.

## Competing interests

The authors declare that they have no competing interests.

## Authors’ contributions

KL carried out the analyses, and drafted and revised the manuscript. RI and BSM designed and RI coordinated the study. RI, DH, JP and BSM helped to prepare and edit the manuscript. All authors read and approved the final manuscript.
